# Regaining Confidence after Stroke (RCAS): a feasibility randomised controlled trial (RCT)

**DOI:** 10.1186/s40814-019-0480-z

**Published:** 2019-07-25

**Authors:** Jane C. Horne, Kate E. Hooban, Nadina B. Lincoln, Pip A. Logan

**Affiliations:** University of Nottingham, School of Medicine, Division of Rehabilitation and Ageing, B floor-Rm98, QMC Campus, Nottingham, NG7 2UH UK

**Keywords:** Feasibility, Confidence, Stroke, Psychological therapies, Group intervention

## Abstract

**Background:**

The Regaining Confidence after Stroke (RCAS) course was designed to facilitate adjustment for people with stroke discharged from rehabilitation. The aim of the trial was to evaluate the feasibility of conducting a randomised trial to compare a RCAS course with usual care. The rates feasibility of screening and recruitment, rates of consent and retention, acceptability of outcome measures and the acceptability and fidelity of the intervention were evaluated.

**Methods:**

Participants with stroke were recruited from hospital databases and community services and randomly assigned to the Regaining Confidence after Stroke (RCAS) course or usual care. The course comprised 11 weekly 2-h sessions with six–eight participants, delivered by two rehabilitation assistants. Carers were invited to attend three of the sessions. Sessions were video recorded. A six-item checklist was developed from the manual content. Each item was rated as met, partially met or not met. Fidelity was assumed if > 75% of the criteria were met. Outcomes were assessed three and six months after randomisation. Semi-structured interviews were conducted using open-ended questions to assess the acceptability of the intervention.

**Results:**

Of 47 participants (mean age 66.9 years [SD 14.9]; 26 men), 22 were randomly allocated to the intervention and 25 to usual care. Participants attended a mean of 8.2 [SD 2.6] out of 11 sessions. Fidelity outcomes suggested that the content corresponded to the manual but further training of the therapist was needed. Interview findings indicated the intervention was acceptable and considered beneficial. At three months, 35 (78%) participants returned questionnaires and 30 (67%) at six months, but only 38(42%) were fully completed.

**Conclusion:**

The results support the feasibility of conducting a randomised trial to evaluate the effectiveness of a RCAS course compared to usual care.

**Trial registration:**

ISRCTN 36330958

**Electronic supplementary material:**

The online version of this article (10.1186/s40814-019-0480-z) contains supplementary material, which is available to authorized users.

## Key messages

- It was feasible to recruit, consent and retain participants into a trial that compared a Regaining Confidence after Stroke course to usual care.
- The trial procedures were acceptable, and the qualitative data suggested the course was perceived as beneficial to stroke survivors.
- The training required to deliver the course depended on the previous experience of the course leader.
- The outcome measures were too long, and shorter versions were recommended.

## Introduction

There are currently 1.2 million people in the UK living with a disability after stroke, with direct costs to the National Health Service of around 1.7 billion [1]. One in five stroke survivors in the UK reports the emotional impact of stroke is hard to deal with [1] and emotional support after a stroke is a common unmet need [2]. Confidence, a component of emotional impact, is important to stroke survivors; they reported ‘finding the best ways to improve confidence’ as a stroke research priority [3]. Low confidence often leads a fear of going out [4], reduced social function [5, 6] and reduced activity, linked to low mood [7]. These factors often impact on a stroke survivor’s ability to adjust and cope with the consequences of stroke. National policy guidelines indicate there is insufficient evidence for psychological therapies that show clinical and cost-effectiveness [8]. If stroke survivors are to be enabled to lead active and meaningful lives after their stroke, this evidence needs to be developed.

Programmes designed to enhance confidence and strengthen the belief in one’s capability have shown to be effective in improving quality of life [9]. Further evidence to confirm these findings is needed, and uncertainty about how best to deliver these interventions is a recognised gap in the literature [9]. The Regaining Confidence after Stroke course was developed by a clinical psychologist working in stroke services to facilitate psychological adjustment after stroke, drawing on a range of psychosocial approaches, including social learning theory [10], behavioural activation [11] and the cognitive theory of depression [12]. The RCAS course aims to address low mood and confidence; therefore, measures of mood, confidence and activity were included to assess outcomes.

The course follows a structured manual and is delivered in groups weekly for 11 weeks. The focus of the course is on adjusting and coping after a stroke, and the content of the published manual includes setting realistic targets, solving common problems and utilising support. The RCAS course was initially delivered by a clinical psychologist [13], but training was provided for other health care professionals and the manual recommends that the course can be delivered by a person who has at least one year of experience of working in stroke services, or a stroke survivor who has previously attended the course. Having two facilitators per group was also recommended. The RCAS manual recommends the course is delivered to groups of between eight and 12 people, fewer if there are additional communication support needs.

Treatment fidelity is the degree to which a treatment is implemented as intended (treatment integrity) and whether the treatment being evaluated differs from other treatments or the control condition (treatment differentiation) [14]. Dumas et al. [15] distinguished between content fidelity, what was done, and process fidelity, the way it was done. Content and process fidelity were therefore examined.

The aim of this study was to determine whether the RCAS course could be evaluated in a randomised controlled trial. The specific questions were as follows:

1. Is it feasible to screen for eligibility, recruit, consent and retain participants with a stroke and their carers, in a randomised controlled trial?
2. Can the RCAS course be delivered with fidelity?
3. Is the course acceptable to stroke participants and their carers?
4. Can outcome measures be collected by post at 3 and 6 months after randomisation?
5. What are the most appropriate outcome measures for use in a future trial?

## Methods

We conducted a parallel group two-arm feasibility randomised controlled trial with 1:1 allocation in a single centre, comparing an 11-week RCAS course in addition to usual care with usual care alone. Ethical approval was received by the National Research Ethics Service Committee East Midlands, Nottingham 1 (12/EM/0319). The design of the study was informed by CONSORT guidance [16], see Additional file 1.

Stroke survivors were identified through three sources:

- A large acute hospital, stroke database. Potential participants were sent invitation letters if they had been discharged in the last two years and were registered with a general practitioner in the geographical area covered by the trial.
- A large community stroke rehabilitation provider sent invitation letters to stroke survivors who had used the service within the previous two years.
- Community stroke service rehabilitation assistants invited potential participants at the point of discharge from routine rehabilitation.

The letters included a tear-off slip with contact details of potential study participants which were returned to the research team in a stamped addressed envelope. An email or phone call was also acceptable.

All responses were followed up by researchers (KH/JS) who screened potential participants for inclusion by telephone. The inclusion criteria were as follows:

- A clinical diagnosis of stroke within two years
- Discharged from all National Health Service rehabilitation therapies
- Not involved in trials of other psychological interventions
- Had not previously attended an RCAS course

Those who met the inclusion criteria were then posted a copy of the patient information sheet (PIS) and visited by a research assistant (JS) to obtain written consent. They were then further assessed for eligibility. The exclusion criteria were as follows:

- Score < 10 on the Barthel index [17] so that participants would be sufficiently independent to cope in a group setting
- Score < 8 on the Sheffield Screening test for Acquired language Disorders [18] so that participants would be able to understand the material presented and take part in group discussions
- Unable to speak English
- Unable to give informed consent

Baseline measures were then administered to those who were eligible for the study by the research assistant. Responses were recorded in a questionnaire booklet by the research assistant or participants could complete the booklet themselves. The following data were collected:

- Participant characteristics, including age, gender and number of months since stroke
- General health information and living arrangements were recorded to ensure that the group leader had sufficient participant information to run the intervention safely.
- Level of psychological distress using the General Health Questionnaire 30-item version (GHQ-30) [19]. This is a measure of general levels of psychological distress which has previously been found to be responsive to the effects of psychological interventions after stroke [20, 21] and in other conditions using similar interventions [22].
- Level of confidence using the 53-item Confidence after Stroke Measure (CaSM) [23]. This measure was chosen as it specifically developed to assess confidence after stroke as there were no previous measures available, but responsiveness to treatment has not previously been assessed.
- Performance in instrumental activities of daily living using the Nottingham Extended Activities of Daily Living scale (NEADL) [24]. This is a commonly used measure of outcome in stroke rehabilitation trials.
- Ability to use appropriate coping strategies using the Coping Orientation for Problem Oriented Experiences [COPE] inventory [25]. This was selected as the focus of the intervention was on teaching coping strategies.

Participants were asked if they would like their carer to be invited to the study, as three sessions were designed to include carers’ attendance. Participant information sheets were sent in advance and carers were visited at home to obtain written consent.

Carers completed a questionnaire booklet including the following:

- Level of carer strain using the Carer Strain Index [26]
- Level of psychological distress using the GHQ-30 [19]

Help with completing questionnaires was offered on request.

### Randomisation

A study ID was allocated to those who gave consent. The recruiting researcher contacted an independent researcher, who held a computer-generated allocation sequence, to determine the group to which the participant had been randomised. This was to ensure allocation concealment from the recruiting researcher. Carers were allocated to the same group as the stroke participant they cared for.

### Control and intervention group

Stroke survivors allocated to both the control and intervention groups had access to clinical services as usual. Typically, usual care after discharge from stroke rehabilitation community services was provided by Stroke Association support groups and social care services.

Stroke survivors allocated to the intervention group were invited to attend a weekly RCAS course for 11 weeks in addition to usual care. When there were a minimum of eight, the participants were allocated to the intervention to form a group and were contacted to start the course. Intervention was delivered in a community venue. A taxi was provided on request. The sessions were delivered by an assistant practitioner (JS). She had worked for more than one year in stroke rehabilitation, with experience of leading stroke therapy groups and was supported by an assistant psychologist (KM) [13]. Carers were invited to attend sessions one, six and eleven, in accordance with the manual.

The sessions followed the published RCAS manual (Townsend 2009) and were the following:

1. You and Your Supporter: Getting to know one another in the group and sharing experiences
2. Problems in Common: Identifying and solving common problems
3. Setting Realistic Targets: How realistic targets can help recovery
4. Setting Realistic Targets-2: Reviewing goals and planning steps to achieve goals
5. Worry: Understanding what worry is, what effect it can have and how to manage it
6. Understanding One Another
7. Other People in Your Life: Identifying people who affect your feelings
8. Gloom: Why people feel depressed after stroke and ways to stop it occurring
9. Living a New Life: Coping with change and building confidence in coping with change
10. Living a New Life: Looking at what is still the same and what is different following the stroke
11. Summing up: Thoughts on the course and talking about the next steps to recovery

Attendance at sessions and reasons for non-attendance were recorded.

To assess the fidelity of the intervention, group sessions were video recorded. A checklist was used to determine the correspondence between the intervention delivered and the manual (content fidelity). Two independent researchers (AL/CS) examined the video data and coded the content. General criteria were marked on a 3-point scale 0 (not met), 1 (partly met) or 2 (fully met). The aspects considered were the following:

1. Outlined aims and running order of the session
2. Kept on topic
3. Kept the atmosphere light
4. Managed the whole group well
5. Dealt with emotional patients in the correct manner
6. Closed well and answered any questions the patients may have

Session-specific criteria were scored as 0 (not met) or 1 (met). An example is shown below:

Session three

1. Begin with progress report on problems identified at the last meeting
2. View “Where are they now?”
3. Share expectations people had of recovery at different points and emphasis that they alter with time
4. View “At work” and “Leisure” and discuss
5. Discuss areas group members want to achieve more in, set task.

If 75% or more of the components were delivered in each session, it was assumed the intervention was delivered as intended [27].

In addition, to assessing the process fidelity, an independent clinical psychologist reviewed the video recordings and made observations in process notes. The videos and process notes were examined by a second clinical psychologist, a consensus was reached and a summary generated.

### Outcomes

At three and six months after random allocation, participants and carers were sent a questionnaire booklet to complete and return by post. The participant booklet included the NEADL, COPE, GHQ-30 and CASM. The carer booklet included the CSI and GHQ-30.

Participants (*n* = 13) in the first two groups receiving the RCAS course were invited to be interviewed by research associates who did not deliver the intervention (KH/KS), prior to the collection of follow up data. Interviews were conducted in the participants’ own home. The interviews were audio recorded and transcribed verbatim. A topic guide was developed from the literature and revised by steering group members, expert stroke rehabilitation researchers, clinicians and a stroke champion.

One key question was asked:

Tell me about what you thought about the Regaining Confidence after Stroke Course?

The aim was to illicit in-depth data and to use minimum prompts so as not to lead participants, and to allow open responses. Prompts were used to guide participants if needed. Topics explored included any positive content, negative content, potential changes to content/study processes, effect on mood, confidence, social identity and the importance of social context.

### Sample size

The target sample size was 60 participants, based on the upper limit recommended for feasibility studies [28]. This number would be sufficient for four RCAS groups with 6–8 participants in each group [13].

### Statistical analysis

Data were analysed using SPSS (version 21). As this was a feasibility study, the main analysis focussed on descriptive statistics which were computed for each group. Response rates for returned questionnaires were calculated separately for each group. Rates of missing data were calculated for each outcome measure.

### Qualitative analysis

Interview data was analysed thematically, using a framework analysis approach [29]. NVivo (V.10) software was used to code the data and to explore emergent themes across the dataset. Understanding participant’s experience of participating in a confidence course was explored. Barriers and facilitators of attending the course were coded, analysed and themed, by the primary researcher (KH). Carers were not interviewed, but participant’s views of carers being invited to attend selected sessions were explored.

## Results

### Recruitment

**Fig. 1:**
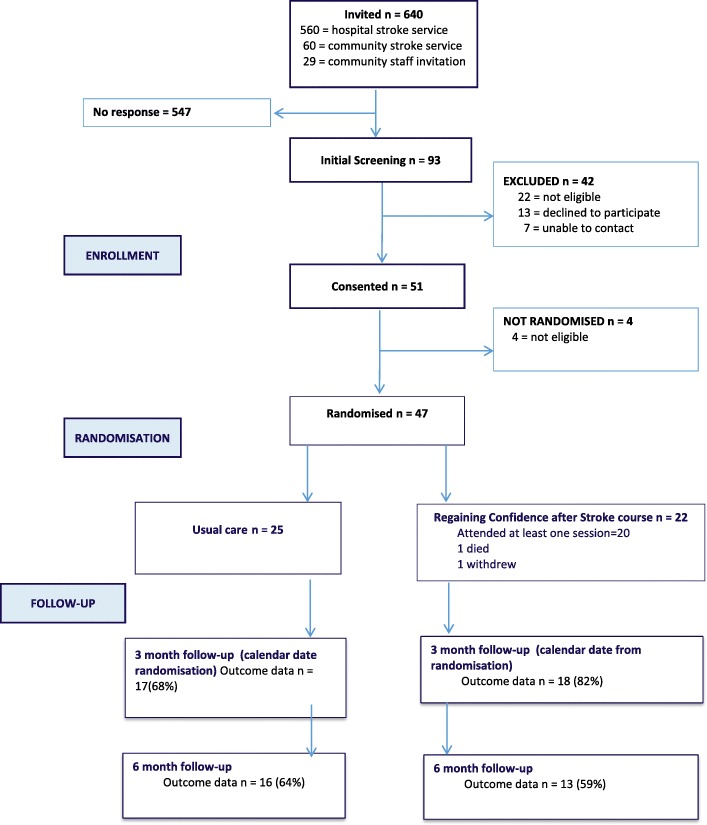
Flow of participants through the study

The flow of participants through the study is shown in the flow diagram (Fig. 1).

Letters were sent to 640 potential participants using three different methods. The lowest response rate was from the acute hospital stroke register (11%) and the best from face-to-face community stroke service invitations (65%). Of the 93 potential participants, 46 (49%) were excluded and 47 (51%) participants agreed to be included. This resulted in 78% of the recruitment target of 60 being achieved in the one year recruitment period (June 2013 to August 2014).

Following randomisation, 22 participants were allocated to the intervention group and 25 to the control group. However, before the intervention began, one died and another did not wish to continue with the study, resulting in outcomes being requested from 20 participants in the intervention group. Eleven carers were recruited, ten women and one man, seven from the intervention group and four from the control group,

Participant characteristics at baseline are presented in Table 1.

**Table 1:** Participant characteristics and baseline scores on questionnaires

Gender	Intervention (*n* = 22)		Control (*n* = 25)	
	*n*	%	*n*	%
Men	11	50	15	60
Women	11	50	10	40
	Mean	SD	Mean	SD
Age in years	69.6	14.1	64.2	15.7
Months since stroke	15.8	11.4	15.8	7.9
Barthel score	18.5	1.8	18.6	1.9
Sheffield Screening Test for Acquired Language Disorders	18.4	1.8	17.9	2.0
Nottingham Extended Activities of Daily Living	13.8	4.9	14.4	5.0
COPE 1 Active coping	12.0	1.8	11.1	3.1
COPE 2 Planning	10.7	2.6	10.1	3.4
COPE 3 Seeking instrumental social support	10.1	3.5	9.8	3.4
COPE 4 Seeking emotional social support	10.6	3.3	9.5	3.9
COPE 5 Suppression of competing activities	10.3	2.1	9.2	3.0
COPE 6 Turning to religion	8.7	3.8	7.6	4.5
COPE 7 Positive reinterpretation and growth	11.3	2.3	11.3	2.7
COPE 8 Restraint coping	10.3	2.7	10.4	2.9
COPE 9 Acceptance	12.5	1.7	12.1	3.2
COPE 10 Focus on and venting of emotions	10.1	2.9	9.2	3.2
COPE 11 Denial	8.6	3.3	7.4	2.6
COPE 12 Mental disengagement	8.8	2.8	9.0	2.3
COPE 13 Behavioural disengagement	8.4	2.5	7.2	3.1
COPE 14 Use of alcohol	4.1	0.4	4.5	1.4
COPE 15 Humour	9.5	4.1	9.1	3.6
General Health Questionnaire -30	26.8	10.9	29.1	13.3
Confidence after Stroke Measure	150.1	17.5	147.2	15.5
Carers	Intervention (*n* = 7)		Control (*n* = 4)	
General Health Questionaire-30	21.0	3.3	35.0	3.6
Carers Strain Index	3.9	3.1	1.7	1.5

The groups were well balanced at baseline.

The mean time between randomisation and starting a group was 37.5 days (SD 21.5, range 6–80). The mean attendance was 7.8 sessions (SD 2.4, range 4–11). Four (20%) intervention participants attended all 11 sessions, but 13 (65%) participants attended 8 or more sessions.

The total number of possible attendances was 220 (20 participants × 11 sessions). There were 77 (35%) missed sessions. Sessions were missed by 16 (80%) participants. The reasons are shown in Table 2. The most common reason for non-attendance was medical reasons.

**Table 2:** Reasons for non-attendance at RCAS sessions

Reason	Participants affected	Number of missed sessions
Unwell	4	24
Medical appointments	3	4
Admitted to hospital	2	13
Holiday	3	5
Other commitments	1	1
Leader cancelled	3	3
No reason given	2	5
Died	1	11
Withdrew from study	1	11

Three out of the seven carers attended all three sessions (43%). Reasons for non-attendance were not collected but non-attendance coincided with non-attendance from the participant.

### Fidelity

Nineteen (43%) of the 44 intervention sessions were recorded.

The results are presented in Table 3.

**Table 3:** Assessment of fidelity of the intervention

Session number	1	2	3	6	8	9	10	11
Percentage of specific criteria met	86	75	100	90	81	100	95	70
Percentage of general criteria met	80	65	85	80	77	70	75	88
Combined percentage of all criteria met	83	70	93	85	79	85	85	76

There were no recordings made of sessions 4, 5 and 7

On the general criteria, topic compliance, light atmosphere, group management and emotional patients managed all achieved 75% or more compliance with the manual. Compliance was lower in outline of aims (53%) and answered questions (42%).

The summary of the process notes produced by the clinical psychologists identified that participants engaged well with the sessions, with the exception of one dominant member in one session. Peer support was evident in each session. Group leaders demonstrated skills and experience that facilitated this engagement and were confident recommending practical solutions to problems. However, there were limitations of psychological knowledge and experience. This was evidenced by missed opportunities to explore thoughts, feelings and emotions, in six out of the eight sessions.

### Outcome data

The return rate for questionnaires is shown in Fig. 1.

The mean frequency of missing data at follow-up is presented in Table 4. This showed that missing items were highest on the COPE and lowest on the NEADL.

**Table 4:** Number of participants with missing items on questionnaires

	Baseline		3 months		6 months	
Participants	Intervention (*n* = 22)	Control (*n* = 25)	Intervention (*n* = 18)	Control (*n* = 17)	Intervention (*n* = 13)	Control (*n* = 16)
	*n* (%)	*n* (%)	*n* (%)	*n* (%)	*n* (%)	*n* (%)
Complete questionnaires	14 (64)	16 (64)	11 (61)	11 (65)	7 (54)	9 (56)
Questionnaires with missing items						
NEADL	1 (5)	0 (0)	5 (28)	2 (12)	7 (54)	5 (31)
COPE	1 (5)	2 (8)	8 (44)	4 (24)	8 (57)	4 (25)
GHQ	4 (18)	4 (16)	5 (28)	1 (6)	5 (36)	3 (19)
CaSM	2 (9)	3 (12)	7 (39)	2 (12)	8 (57)	5 (31)
Carer	Intervention (*n* = 7)	Control (*n* = 4)	Intervention (*n* = 7)	Control (*n* = 4)	Intervention (*n* = 7)	Control (*n* = 4)
Complete questionnaires	6 (86)	4 (100)	6 (86)	2 (50)	6 (86)	4 (100)
Questionnaires with missing items						
GHQ-30	1(14)	0 (0)	0 (0)	0 (0)	1 (14)	0 (0)
CSI	0 (0)	0 (0)	1(14)	2 (50)	0 (0)	0 (0)

*NEADL* Nottingham Extended Activities of Daily Living, *COPE* Coping Orientation for Problem Orientated Experiences, *GHQ* General Health Questionnaire, *CaSM* Confidence after Stroke Measure, *CSI* Carer Strain Index

The outcomes at three and six months are shown in Table 5.

**Table 5:** Comparison of outcomes on questionnaire measures

	3 months					6 months				
	Intervention (*n* = 18)		Control (*n* = 17)		Difference (95%CI)	Intervention (*n* = 13)		Control (*n* = 16)		Difference (95% CI)
	Mean	SD	Mean	SD		Mean	SD	Mean	SD	
Nottingham Extended Activities of Daily Living	12.9	6.1	14.6	6.3	− 2.3 to 6.0	13.5	5.8	14.3	6.3	− 3.6 to 5.2
COPE 1 Active coping	10.0	2.8	11.2	4.4	− 1.3 to 3.7	8.2	2.6	9.9	3.4	− 0.7 to 4.0
COPE 2 Planning	8.6	3.0	10.7	4.9	− 0.7 to 4.9	9.4	3.4	9.1	4.1	− 3.2 to 2.6
COPE 3 Seeking instrumental social support	9.1	2.9	9.3	3.9	− 2.1 to 2.5	8.9	4.4	8.8	3.9	− 3.3 to 3.1
COPE 4 Seeking emotional social support	9.4	3.8	10.2	3.8	− 1.8 to 3.4	8.3	4.4	9.0	3.6	− 2.3 to 3.7
COPE 5 Suppression of competing activities	9.0	3.0	7.5	3.1	− 3.6 to 0.6	8.7	3.7	7.6	2.7	− 3.5 to 1.3
COPE 6 Turning to religion	8.7	4.9	7.4	4.7	− 4.6 to 2.0	7.3	4.2	7.0	4.6	− 3.7 to 3.1
COPE 7 Positive reinterpretation and growth	9.1	3.2	11.4	3.8	− 0.1 to 4.7	9.6	3.4	10.2	3.7	− 2.1 to 3.3
COPE 8 Restraint coping	9.7	2.2	9.9	3.2	− 1.7 to 2.1	9.6	3.2	9.6	2.8	− 2.3 to 2.3
COPE 9 Acceptance	11.1	2.3	12.2	3.7	− 1.0 to 3.2	11.2	2.9	12.7	3.4	− 0.9 to 3.9
COPE 10 Focus on and venting of emotions	9.8	2.7	8.7	3.7	− 3.3 to 1.1	10.33	3.1	8.3	3.1	− 4.4 to 0.3
COPE 11 Denial	7.3	3.2	7.0	2.9	− 2.4 to 1.8	7.8	3.1	5.6	1.8	− 4.1 to 0.3
COPE 12 Mental disengagement	8.9	2.3	9.0	3.8	− 2.0 to 2.2	8.1	2.5	7.8	3.0	− 2.4 to 1.8
COPE 13 Behavioural disengagement	7.7	2.4	7.0	2.8	− 2.5 to 1.1	7.2	3.5	7.3	3.3	− 2.5 to 2.7
COPE 14 Use of alcohol	4.0	0.0	5.2	3.4	n/a	4.0	0.0	4.9	1.9	n/a
COPE 15 Humour	7.1	3.5	8.6	4.7	− 1.3 to 4.3	8.0	3.7	8.7	3.0	− 1.8 to 3.2
General Health Questionnaire-30	32.5	12.5	36.1	19.9	− 7.8 to 15.0	36.6	21.3	30.4	16.6	20.6 to 8.2
Confidence after Stroke Measure	144.2	18.3	147.0	25.9	− 12.5 to18.1	135.1	15.3	150.4	25.2	− 1.01 to 29.5
Carers	Intervention (*n* = 7)		Control (*n* = 4)							
General Health Questionnaire 30	21.8	7.6	25.0	14.8	− 11.8 to 18.2	25.0	7.0	30.0	16.1	− 10.5 to 20.5
Carer Strain Index	3.8	1.5	2.0	0.0	n/a	5.5	3.1	5.5	4.8	− 5.3 to 5.3

The outcomes at 3 months were used to calculate the required sample size for a future trial. Given these differences between the groups, with a power of 80% and a significance level of 5%, 378 participants are required using the GHQ, 520 using the NEADL and 1342 using the CASM.

### Acceptability

Eight (73%) participants consented to be interviewed. The mean length of the interviews was 24.47 min (SD 19.83). Six key themes were identified: group dynamics, group leader, group delivery, mood and confidence, perceived group benefits and potential barriers.

#### Group dynamics

A difference of opinion on the optimum group size was noted. One participant described ten as being preferable to small groups, whilst others preferred eight or fewer. Benefits for both large groups and small groups were described. Strong peer support within the group created opportunities to talk through problems and feelings with people who have experienced similar challenges.

To hear other people, I found that so supportive. It was somewhere to go and meet people. We could sit and tell each other. (1005)

We were strangers then we got to know each other...you’d blend in like a jigsaw puzzle.....It reminded me of a family gathering. (1004)

However, gender imbalance was described as being an issue for one man, as he was the only male in the group.

#### Group leader

The group leader was described in positive terms, professional, expert and good at her job. Knowledge of stroke and experience of leading a group appeared to be vital components of positive experiences being described by the group.

(She is) a real good man manager....she really knows how to work people.....she knows how to put a question to draw the best out of you. (1006)

She were brilliant talking to us, putting us minds at rest in lots of ways. She gave you confidence. (1026)

Participants described the course leader’s knowledge and trust in her ability. These key factors appeared to contribute to the groups’ acceptability.

#### Group delivery

The frequency and length of sessions were not explored in depth; however, one described the frequency as appropriate, and another would have preferred a longer course:

The content of the course was described in positive terms, such as ‘helpful’, ‘refreshing ‘and ‘varied’.

I liked the idea of giving us some things to think about for the week and writing it down in a folder then dissecting it the following week. (1006)

Too much discussion was not appreciated by one participant.

We all compared notes each week-it was alright sitting talking about things but I wanted to get on and do things, for myself. (1019)

The content of the course was well received, and participants described overall individual benefit. Practical issues, such as the taxi not arriving, impacted negatively on participant experience.

#### Confidence and mood

There were positive effects on both confidence and mood.

It gave you confidence. Confidence when people tell you what you can do and somebody tells you how to take that step. (1004)

I’d lost all my confidence but I’m gradually getting it back- I just do it one step at a time. (1005)

However, one participant stated:

I didn’t feel any more confident when I’d been. It didn’t really do anything for me. (1019)

#### Perceived benefits

There was a reported sense of loss when the groups ended, and positive language was used when describing the benefits: ‘enjoyed it’, ‘well worth going to’, ‘brilliant course’ and ‘it was all amazing’. Participants reported increased activity as a result of the course.

It made me think I’m going to go out for a walk and I’ve been doing it, going out on my own. (1013)

Going to those classes opened up a big world for me, it has given me a hell of a lot to aim for. I aimed for a goal each time and I have achieved quite a lot up to now. (1005)

The educational benefits were described:

You were learning whilst you was talking. (1004)

It helped me think about what causes a stroke’ and ‘it was really interesting finding out what help is there. (1005)

Reflecting on this learning, ‘writing down feelings’ and ‘thinking differently about things’ were described.

#### Key facilitators

Group cohesion and peer support were described as being factors in motivating stroke survivors to attend and return to the group sessions. Feeling part of something as opposed to feeling isolated helped members feel they were giving as well as receiving. Easy accessibility of a community venue facilitated attendance. The knowledge of stroke demonstrated by the group leader was considered an essential skill. Natural ability to facilitate a group was observed and encouraged participation in the sessions. Articulating not wanting the course to end suggested participants found the experience acceptable.

#### Potential barriers

The key barrier was transport problems to the venue, and this may have impacted on attendance. Gender balance, personality and levels of disability influenced group dynamics.

## Discussion

The primary aim was to evaluate the feasibility of undertaking a randomised trial, comparing the Regaining Confidence after Stroke Course with usual care. The trial was feasible but some aspects could be changed in a future trial.

It was feasible to recruit participants with stroke from the community. Face-to-face recruitment from a large community stroke service resulted in better recruitment than postal invitations. Evidence from recent studies [21–23] suggests mistrust, failing to see personal benefits and travel time to participate, and led to lack of engagement in trials. A face-to-face approach is more likely to be successful. Telephone reminders, as a follow-up to postal invitation, may also have a positive effect on recruitment [30]. In addition, good communication methods and training of recruiters [31] may increase recruitment rates. Therefore, a future trial would rely on personal contact for recruitment, if possible, with telephone reminders for any postal invitations.

The intervention was successfully delivered, and results suggested that it might benefit people with a stroke. The typical NHS provision for self-management group interventions is six weekly sessions [32]. Although a recent systematic review reported the dose of self-management programmes ranged from four weeks to six months [9], the authors highlighted a gap in knowledge of the optimal timing of such interventions. A six week course could be more aligned to the current NHS provision. Qualitative findings suggested that evaluating a shorter course that would reduce costs may be appropriate. However, there is no evidence to conclude whether a shorter course would have an equivalent benefit. Shortening the course to six sessions would need to be explored prior to an evaluation of effectiveness.

The study aimed to recruit a minimum of eight members to each group as eight–12 were recommended in the manual, less with additional support needs. However, two dropouts in groups one and two, prior to starting the course, and time limitations at the end of the study resulted in a minimum of six members in two groups. Size was explored within the qualitative data, and the findings indicate that the smaller groups were acceptable. Attendance at sessions was good and comparable to other group psychological interventions [33, 34]. Health issues, medical appointments, hospital admissions and problems with travel impacted on attendance. The qualitative data supported the acceptability of the intervention: participant’s reported positive experiences of attending the group.

Only 11 (23%) participants had carers willing or able to attend sessions, but these carers showed good attendance. Interviews with carers would have elicited more details of the acceptability of the intervention for carers.

Assessment of the content fidelity of the intervention suggested good compliance with the content of the manual. However, not all sessions were recorded, for reasons that are unclear. Further training of the course leader may be necessary to ensure good compliance with general aspects of delivering the group. The evaluation of process fidelity highlighted that whilst the group leader was confident recommending practical solutions to problems, she lacked skills in recognising and addressing psychological and emotional issues. This has previously been identified as a problem for those working in stroke services [35] and highlights the need for specific training in psychological problems after stroke. The manual was developed by an experienced clinical psychologist, and the RCAS course has often been delivered by clinical psychologists in stroke services. Because they are already well trained to recognise and deal with emotional problems, this aspect of the intervention may not have been adequately emphasised in the manual when recommending that the course can be delivered by other health care professionals. It is therefore important that adequate initial skill training and ongoing supervision are embedded in future trials.

There was a high loss to follow-up over time. Whilst these rates are similar to some stroke rehabilitation trials [36, 37], strategies to improve completion rates are recommended for future trials. These include telephone prompts for those who fail to return questionnaires and face-to-face visits [38]. In addition, it would be possible to offer the option of completing questionnaires online rather than returning them in the post.

The outcome measures were assessed for appropriateness. The COPE had 60 items and demonstrated the most missing data. Although the items seem appropriate, this measure was problematic to analyse as it has 15 separate domains. Further work on the psychometric properties of the scale is needed before it is used as an outcome measure. It might however be useful as a baseline to highlight positive and negative coping strategies, which could be discussed during group sessions. The CaSM was also lengthy (53 items) and was used because it was, at the time of the study, the only known measure of confidence that was specific to stroke. However, a shortened 27-item version is now available [23] with evidence of validity and reliability.

The GHQ-30, had the least missing data; however, there is a shorter version, the GHQ-12, that has comparable psychometric properties without losing sensitivity [39]. The shorter version includes an item, important for this study: ‘Have you recently been losing confidence in yourself?’ The results suggested that using the shorter versions of the GHQ and CaSM [23] may improve the completeness of outcome data. The NEADL and CSI demonstrated good completion rates and could be used in a future trial.

The qualitative study was limited to interviews with the intervention group, exploring barriers and facilitators of attending the group. This component of the study would have been strengthened by interviewing both groups and exploring the acceptability of study procedures.

## Conclusion

This study provides evidence that evaluating the Regaining Confidence after Stroke course in a future trial is feasible. However, some aspects of the design need modifying. Face-to-face recruitment should be used whenever possible. A shorter RCAS course may be better in line with current NHS provision and therefore more likely to be implemented if found to be effective. Postal outcome data collection could be improved by the availability of help with completing questionnaires if needed. The GHQ-12 would be recommended as a primary outcome in a definitive trial as it will fit with existing meta-analyses and has an established track record. The CaSM-27 and NEADL would be suitable secondary outcome measures to identify any benefits of the intervention. Training of group facilitators in low-intensity psychological interventions, with regular supervision, ideally from a clinical psychologist, would enhance the delivery of the course.

The participants reported benefits of attending the sessions and indicated acceptability of the course. Implementing these recommendations would make it feasible to conduct a phase II trial to assess the effectiveness of the intervention and would build the evidence for group psychological therapies after stroke.

## Additional file

Additional file 1: Consort Checklist. (DOC 226 kb)

## Data Availability

Datasets used and analysed during the current study are available from Pip.logan@nottingham.ac.uk or the corresponding author on reasonable request.

## Abbreviations

CaSM: Confidence after Stroke Measure
COPE: Coping Orientation for Problem Oriented Experiences
CSI: Carers Strain Index
GHQ-30: General Health Questionnaire (30 item)
NEADL: Nottingham Extended Activities of Daily Living
NHS: National Health Service
NIHR: National Institute for Health Research
NRES: National Research Ethics Service
RCAS: Regaining Confidence after Stroke
RCT: Randomised Ccontrolled Ttrial
